# Postsynaptic Stability and Variability Described by a Stochastic Model of Endosomal Trafficking

**DOI:** 10.3389/fncel.2019.00072

**Published:** 2019-02-26

**Authors:** Taegon Kim, Keiko Tanaka-Yamamoto

**Affiliations:** ^1^Center for Functional Connectomics, Korea Institute of Science and Technology (KIST), Seoul, South Korea; ^2^Division of Bio-Medical Science & Technology, KIST School, Korea University of Science and Technology, Seoul, South Korea

**Keywords:** endosome, stochastic model, postsynapse, long-term plasticity, endosomal sorting

## Abstract

Neurons undergo dynamic processes of constitutive AMPA-type glutamate receptor (AMPAR) trafficking, such as the insertion and internalization of AMPARs by exocytosis and endocytosis, while stably maintaining synaptic efficacy. Studies using advanced imaging techniques have suggested that the frequency of these constitutive trafficking processes, as well as the number of AMPARs that are involved in a particular event highly fluctuate. In addition, mechanisms that trigger some forms of synaptic plasticity have been shown to include not only these processes but also additional fluctuating processes, such as the sorting of AMPARs to late endosomes (LEs). Thus, the regulation of postsynaptic AMPARs by the endosomal trafficking system appears to have superficially conflicting properties between the stability or organized control of plasticity and highly fluctuating or stochastic processes. However, it is not clear how the endosomal trafficking system reconciles and utilizes such conflicting properties. Although deterministic models have been effective to describe the stable maintenance of synaptic AMPAR numbers by constitutive recycling, as well as the involvement of endosomal trafficking in synaptic plasticity, they do not take stochasticity into account. In this study, we introduced the stochasticity into the model of each crucial machinery of the endosomal trafficking system. The specific questions we solved by our improved model are whether stability is accomplished even with a combination of fluctuating processes, and how overall variability occurs while controlling long-term synaptic depression (LTD). Our new stochastic model indeed demonstrated the stable regulation of postsynaptic AMPAR numbers at the basal state and during LTD maintenance, despite fast fluctuations in AMPAR numbers as well as high variability in the time course and amounts of LTD. In addition, our analysis suggested that the high variability arising from this stochasticity is beneficial for reproducing the relatively constant timing of LE sorting for LTD. We therefore propose that the coexistence of stability and stochasticity in the endosomal trafficking system is suitable for stable synaptic transmission and the reliable induction of synaptic plasticity, with variable properties that have been observed experimentally.

## Introduction

To stably maintain synaptic transmission, stable regulation of the number of postsynaptic receptors is crucial. However, postsynaptic receptors are not static even under basal conditions, but are rather dynamic (Bredt and Nicoll, 2003; Choquet and Triller, 2003; Lau and Zukin, 2007; Luscher et al., 2011; Lu and Roche, 2012). In particular, the dynamics of AMPA-type glutamate receptors (AMPARs) at excitatory synapses have been well studied. Postsynaptic AMPARs constantly move by lateral diffusion along the plasma membrane (Choquet and Triller, 2003). In addition, postsynaptic AMPARs are internalized by endocytosis and are inserted back into the plasma membrane by exocytosis. Therefore, dynamic degrees of freedom in postsynaptic AMPAR regulation arise from several trafficking processes that AMPARs undergo in the cytosol. In neurons as well as other cells, intracellular trafficking of receptors is mediated by intracellular membrane-bound compartments, namely, endosomes, so that the regulation of the endosomal trafficking pathway at least in part determines receptor trafficking processes, such as recycling, degradation, and the supply of receptors (Bacaj et al., 2015; Bredt and Nicoll, 2003; Brown et al., 2005, 2007; Ehlers, 2000; Fernández-Monreal et al., 2012; Gerges et al., 2004; Lu and Roche, 2012; Matsuda et al., 2013; Petrini et al., 2009). Such regulation occurs constantly to maintain a basal level of postsynaptic AMPARs, and is altered by input stimuli that trigger postsynaptically expressed synaptic plasticity.

Two crucial questions in postsynaptically expressed long-term synaptic plasticity are how cellular components are orchestrated to change the number of postsynaptic AMPARs and how this change in AMPAR number is maintained. Previously reported models of cerebellar long-term depression (LTD), which assume an imbalance between endocytosis and exocytosis by a positive feedback molecular switch (Tanaka and Augustine, 2008; Ogasawara and Kawato, 2009b) can answer the former question. However, these models cannot answer the latter question, because this molecular switch is experimentally suggested to be turned off or lose its effect with time, and hence the imbalance would not last as long as the plasticity is maintained (Kim and Tanaka-Yamamoto, 2013). Thus, to answer this latter question, an extended model is required, which includes another regulatory pathway that comes into the picture after the positive feedback switch loses its effect.

On the other hand, previous studies indicated that endosomal trafficking in the postsynaptic cytosol is necessary for long-term plasticity (Ehlers, 2000; Gerges et al., 2004; Brown et al., 2005, 2007; Petrini et al., 2009; Fernández-Monreal et al., 2012; Matsuda et al., 2013; Bacaj et al., 2015). In particular, our recent work on cerebellar LTD demonstrated that another switch working after the positive feedback molecular switch loses its effect, is likely to be a member of the endosomal trafficking pathway (Kim et al., 2017). The stimulation triggering LTD at synapses between parallel fibers (PFs) and Purkinje cells (PCs) activates a positive feedback loop of protein kinase C (PKC) and mitogen-activated protein kinase (MAPK; Tanaka and Augustine, 2008). This loop enhances the internalization of AMPARs by endocytosis, which results in an imbalance between endocytosis and exocytosis as mentioned above, and in LTD expression. However, the activity of this loop is not required to maintain LTD in the long term. In our previous study, we optogenetically interfered with endosomal trafficking of cargo from early endosome (EE) to late endosome (LE) at different time points, and observed that LTD was impaired when the LE sorting was blocked at 8–23 min after LTD induction. The deterministic model mimicking characteristics of the Rab5-Rab7 conversion switch, which is an essential process for sorting from EE to LE (Rink et al., 2005; Poteryaev et al., 2010), successfully described long-lasting LTD under the short-lasting imbalance between endocytosis and exocytosis due to the diminished effect of the PKC-MAPK positive-feedback loop. In addition, we analyzed individual examples of experimental results and found two distinct responses to the optogenetic interference of LE sorting at the same time points, suggesting different timing of sorting in individual examples. Our results demonstrated that the timing of sorting was partially determined by the speed of LTD expression, and our deterministic model further predicted that another parameter determining the timing is the variable threshold of the Rab5-Rab7 conversion switch. However, experimental observation of endosomal trafficking suggests the existence of other candidates that may be involved in creating the variability in timing of sorting, yet their involvement has not been tested to date.

In this study, we introduced the experimentally known stochasticity of each endosomal trafficking process including the sorting switch from EE to LE, to create a stochastic postsynaptic LTD model. This simplified trafficking model only contains essential dynamic processes but reliably reproduces the time course of LTD with high variability in the timing of sorting AMPARs from EE to LE. Our results from this example system of cerebellar LTD reconfirm the idea that endosomal trafficking is a crucial cellular pathway for long-term plasticity (Ehlers, 2000; Brown et al., 2005, 2007; Fernández-Monreal et al., 2012; Matsuda et al., 2013) and support that the variability in observable parameters arises from the innate stochasticity of each microprocess.

## Materials and Methods

### Model Construction—Compartments

**Figure 1:**
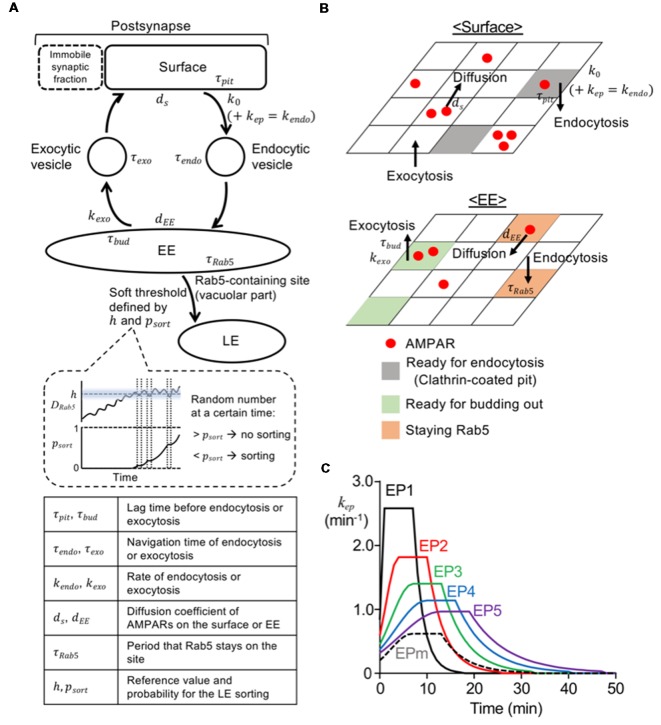
Model construction and various endocytosis profiles. **(A)** Diagram of overall structure of current model. The parameters (*τ*, *k*, *d*, *h*, *p*_sort_) are explained at the bottom, and values of these parameters used in the model are shown in Table 1. The panel enclosed with a dashed line in the middle shows an illustration presenting an increase in *p*_sort_ depending on the duration of *D*_Rab5_ above the *h*. The sorting occurred, when a random number was below *p*_sort_ at a certain time. **(B)** Diagram of surface (top) and early endosome (EE; bottom) compartment. Red filled circles represent AMPA-type glutamate receptors (AMPARs) and they could diffuse laterally on surface or EE. Gray, green, and orange parts are the sites ready for endocytosis from the surface, the site ready for exocytosis from the EE, and Rab5 positive EE sites, respectively. **(C)** Time course of the rate for stimulus-dependent endocytosis applied in the simulation. Long-term synaptic depression (LTD) inducing stimuli were represented by endocytic profiles, EP1–5 (solid lines; EP1—black, EP2—red, EP3—green, EP4—blue, EP5—purple), and mild stimulus was described by EPm (black dashed line).

The deterministic model that we previously created (Kim et al., 2017) contained all the essential compartments to describe endosomal trafficking as well as lateral diffusion on the synaptic and extrasynaptic surface. The stochastic model in this present study also utilized the same essential compartments (Figure 1A), but the detailed structures of two compartments were modified. First, the extrasynaptic fraction originally considered in the deterministic model was simplified and treated as part of the mobile synaptic fraction (*S*_m_), so that the surface compartment (Figure 1B) was basically composed of only the territories of *S*_m_. As was the case in the previous model, the sum of *S*_m_ and the fixed immobile synaptic fraction (*S*_im_) represents the number of postsynaptic AMPARs. The surface compartment was assumed to be a square lattice made of 50 × 50 homogeneous sites where AMPARs can freely diffuse (Figure 1B). Second, the EE was also simplified as a square lattice consisting of the same number of sites as the surface compartment (Figure 1B). Two subcompartments of the EE, i.e., one for recycling and one for being sorted to LE (vacuolar part), were introduced. Each EE site could contain AMPARs and a single Rab5 molecule, and the all Rab5-containing sites were assumed to be the vacuolar portion. Each site on the surface or the EE was able to contain an unlimited number of AMPARs, but highly clustered AMPARs in a site were not observed during any of the simulations in this study.

### Model Construction—Trafficking Processes of Endocytosis and Recycling

The movement of each AMPAR starting from the surface compartment can be described first by endocytosis and then by either recycling or sorting to LE (Figure 1A). For model construction of endocytosis and recycling processes, the surface compartment was constantly exposed to endocytosis and exocytosis, and AMPARs on the surface were able to diffuse with the diffusion coefficient *d_s_*. Each lattice site was assumed to randomly form a clathrin-coated pit with a rate of *k*_0_, under no stimulus (Wang and Linden, 2000). To mimic the application of stimuli, the stimulus-representing endocytosis profile (EP, *k*_ep_ in Figure 1C, see “Simulation” section) was added to *k*_0_, which eventually formed the time-dependent *k*_endo_ (Tao-Cheng et al., 2011). The clathrin-coated pit was endocytosed after a lag time of *τ*_pit_. AMPARs at these sites were internalized upon endocytosis of these sites, and they hence existed on the endocytic vesicles. Each endocytic vesicle with or without AMPARs arrived at the EE after a navigation time of *τ*_endo_, and immediately fused to a randomly chosen site on the EE, once it arrived. Upon the fusion of vesicles, Rab5 was assumed to be recruited to the site on the EE, and consequently AMPARs on the vesicles were colocalized with Rab5. Whereas Rab5 remained on the fusion site during a period termed *τ*_Rab5_, AMPARs diffused on the EE with the diffusion coefficient *d*_EE_. Rab5-free EE sites could bud out with a rate of *k*_exo_, and then became exocytic vesicles after a period termed *τ*_bud_. Exocytic vesicles traveled toward the surface compartment during a period of *τ*_exo_ and then immediately fused at a random site on the surface compartment. Values of *τ*_pit_ and *τ*_bud_ were drawn from a uniform random distribution, and values of *τ*_endo_ and *τ*_exo_ were drawn from a Gaussian random distribution.

### Model Construction—Rab5 Accumulation and Sorting From EE to LE

As briefly mentioned above, in our model, Rab5-positive EE sites represent the vacuolar portion of the EE. According to the experimental results (Rink et al., 2005; Poteryaev et al., 2010) and modeling study (Del Conte-Zerial et al., 2008), the kinetics of Rab5 accumulation appear to follow the kinetics of autocatalysis, which was introduced by the simplified equation in the previous deterministic model (Kim et al., 2017). In the current model, Rab5 accumulation was simulated by adjusting the lifetime of Rab5 in an EE site (*τ_Rab5_*) to be similar to the kinetics of formation of the vacuolar portion in the previous model, using the equation shown below.

$$\tau_{\text{Rab5}}(D_{\text{Rab5}})=\frac{KD_{\text{Rab5}}(C-D_{\text{Rab5}})}{1+e^{-\frac{D_{\text{Rab5}}-a}{b}}}$$

where *D_Rab5_* was the fraction of Rab5-positive sites in the EE, representing Rab5 accumulation. The numerator term represents autocatalysis with a limiting factor whereas the denominator term further shaped the rising kinetics. Thus, the coefficients *a* and *b* were the shape adjusting parameters, *C* was the limiting level of accumulation, and *K* was the scaling parameter. The newly updated *τ*_Rab5_ was applied to the newly arrived Rab5 but did not affect the already existing Rab5.

In our present model, a soft threshold was assumed for the threshold of sorting from EE to LE. The soft threshold was defined by two parameters, i.e., a reference value (*h*) and sorting probability (*p*_sort_). The *h* value was drawn from a Gaussian random distribution. The *p*_sort_ value exponentially increased depending on the total duration of  *D*_Rab5_ above the *h* (Figure 1A), and was described by the following equation:

$$p_{\text{sort}}(t)=\{\begin{array}{cc} 0, & t\leq t_{exc}\\ 1-min\{e^{-(t-t_{exc}-\tau_{ud})/\tau_{s}},1\}, & t>t_{exc} \end{array}$$

where *t_exc_* was the first moment of *D*_Rab5_ reaching *h*, and *τ*_ud_ was the total period of *D*_Rab5_ below the *h* after *t*_exc_. By tuning *τ*_s_, the level of softness of the threshold could be adjusted. Sorting from EE to LE occurred when a random number drawn from the uniform distribution on the interval [0,1] was below *p*_sort_ at a certain time.

### Simulation

All simulation procedures were performed by the built script on MATLAB (Mathworks, Natick, MA, USA). The coefficient values used here are shown in Table 1. The time step was 0.1 s, and the entire simulation was repeated 100 times. To set the numbers of AMPARs on different compartments, we first assumed that there were 150 AMPARs on the surface and 100 AMPARs on the EE, and ran the trafficking through endocytosis and recycling until the numbers in both compartments became stable. We then used averaged numbers from 5 min as the initial number of AMPARs on the surface, the EE, and endocytic and exocytic vesicles for the simulation. The external stimulus, which enhanced endocytosis, was represented by several types of EPs (Figure 1C). As in the previous deterministic model (Kim et al., 2017), EPs were described by a piecewise-defined concave function, which consists of a Gaussian rising (0 ≤ *t* < *t*_peak_ − 3), a steady value (*t*_peak_ – 3 ≤ *t* < *t*_peak_ + 3), and an exponential decay(*t*_peak_ + 3 ≤ *t*). The peak timing (*t*_peak_) of the LTD-inducing stimuli EP1–5 (Figure 1C; solid lines) were varied to describe the different speeds of LTD expression, yet integration along the entire stimulation time was tuned to be the same to conserve the magnitude of the stimulus. To describe mild stimuli, EPm (Figure 1C; black dashed line) was assumed to have the same peak timing with EP3, but its integration was set to be significantly lower.

**Table 1:** Parameter values used in the simulation.

Parameters	Values	Note
*S*_im_	40	
*k*_0_	1.2 min^−1^	
*τ*_pit_ ~ *U (n, m)*	*U* (1 s, 2 s)	Drawn from uniform distribution of interval *[n, m]*
*τ*_endo_ ~ *N* (*μ*, *σ*)	*N* (4 min, 1 min)	Drawn from Gaussian random distribution of mean, *μ*, and standard deviation, *σ*.
*a*	0.2	
*b*	0.02	
*C*	1.1	
*K*	20	
*d*_EE_	0.05 μm^2^·s^−1^	
*k*_exo_	1.0 min^−1^	
*τ*_bud_ ~ *U (n, m)*	*U* (1.5 s, 2.5 s)	Drawn from uniform distribution of interval *[n, m]*
*d*_s_	0.05 μm^2^·s^−1^	
*τ*_exo_ ~ *N* (*μ*, *σ*)	*N* (2 min, 0.5 min)	Drawn from Gaussian random distribution of mean, *μ*, and standard deviation, *σ*.
*h* ~ *N* (*μ*, *σ*)	*N* (0.4, 0.02)	Drawn from Gaussian random distribution of mean, *μ*, and standard deviation, *σ*.
*τ*_s_	0.38 min	

## Results

### Stable Maintenance of Postsynaptic AMPAR Number

**Figure 2:**
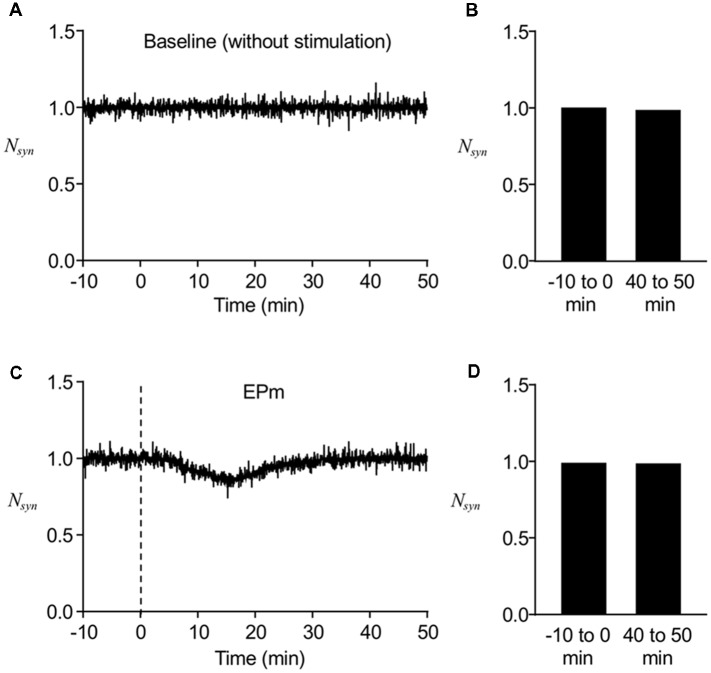
Stable regulation of AMPAR numbers in a postsynapse. **(A)** A representative time course of normalized postsynaptic AMPAR numbers (*N_syn_*) without stimulation. **(B)** Averaged *N*_syn_ comparison between −10 to 0 min and 40–50 min. **(C)** A representative time course of *N*_syn_ with mild stimulus, EPm. **(D)** Averaged *N*_syn_ comparison between period before (−10 to 0 min) and 40–50 min after the start of EPm latter of which is the time when EPm is supposed to be phased out. Dashed lines in this and subsequent figures show the time when the stimulation (endocytic profiles) started.

We first determined whether the stochastic model we built reproduces the stable maintenance of postsynaptic AMPAR number. Without any perturbing stimulus, the normalized number of postsynaptic AMPARs (*N_syn_*) was mostly conserved over time, although there were fluctuations during short time periods (Figure 2A), which were confirmed by comparing the average *N*_syn_ at earlier time points (−10 to 0 min) with that at later time points (40–50 min; Figure 2B). To confirm the stability of the model system under a weakly perturbing stimulus, EPm (Figure 1C) was applied at *t* = 0, which altered the time course of *N*_syn_ and led to a decrease from the baseline (−10 to 0 min) for a finite time period (Figure 2C). With time, *N*_syn_ was recovered to the same level as the baseline (Figures 2C,D). These results showed that the newly built stochastic trafficking model was able to describe the stable regulation of the number of postsynaptic AMPARs, despite its rapid fluctuation.

### Rab5 Accumulation in the EE

**Figure 3:**
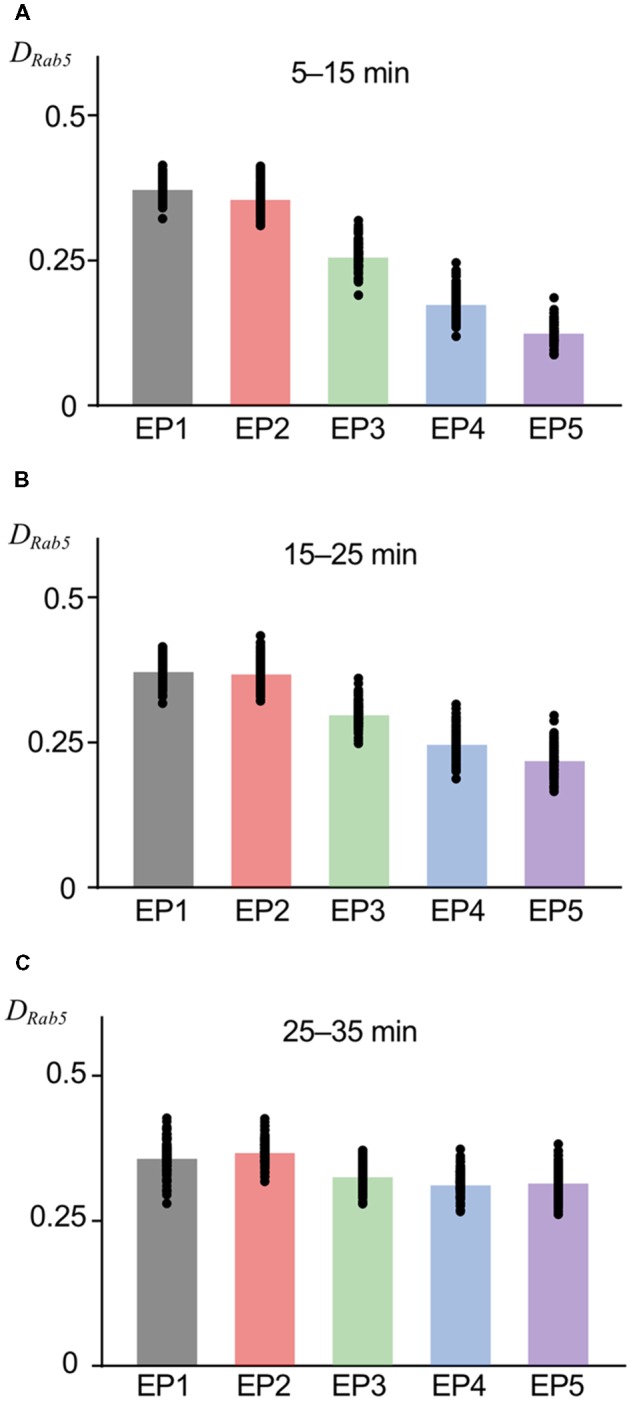
Rab5 accumulation in EE at various timing. The fraction of Rab5 positive sites in EE (*D_Rab5_*) at 5–15 min **(A)**, 15–25 min **(B)**, and 25–35 min **(C)**. Black filled circles show the value from the individual simulation, and bar graphs show averaged values, in this figure as well as subsequent figures showing colored bar graph with black filled circles.

As previous experimental and theoretical studies on intracellular trafficking have indicated (Huotari and Helenius, 2011; Vilar and Saiz, 2011), an essential function of EEs, i.e., the sorting from EE to LE, can be described by the Rab5-Rab7 conversion switch. Regarding the mechanism of this switch, the autocatalytic accumulation of Rab5 in the EE is crucial, which was deterministically modeled in a previous model (Kim et al., 2017). We conserved the autocatalytic accumulation of Rab5 with a competitive degradation term (Del Conte-Zerial et al., 2008); however, before investigating AMPAR trafficking during the switch-on, we tried to confirm reliable Rab5 accumulation in the EE using the current stochastic model. For this purpose, we used other endocytosis profiles (EP1–EP5), which are considered to be triggered by stronger stimuli, and Rab5 accumulation was measured by calculating the fraction of Rab5-positive sites in the EE (*D_Rab5_*). As shown in Figure 1C, EP1–EP5 had different endocytosis speeds, yet had similar magnitudes of stimulation, as seen in the conserved integration along the entire stimulation time. For the early period (5–15 min) after the start of the stimulus-representing endocytosis profile, concentrated endocytosis within a short period (EP1) showed a higher *D*_Rab5_ (Figure 3A). As time went by, the difference in *D*_Rab5_ between focused endocytosis (EP1) and dispersed endocytosis (EP5) became smaller, as seen during 15–25 min and 25–35 min (Figures 3B,C). These results indicated that Rab5 accumulation proportionally followed the time course of the endocytosis profile until a certain saturation limit of the accumulation, as shown in the past experimental results (Rink et al., 2005; Poteryaev et al., 2010). Thus, we confirmed the ability of the current model to regenerate Rab5 accumulation, as expected previously. Needless to say, individual examples (shown by filled circles in Figure 3 as well as the following figures) of Rab5 accumulation varied due to the properties of the stochastic model.

### Proportional Accumulation of Internalized AMPARs With Higher Variability

**Figure 4:**
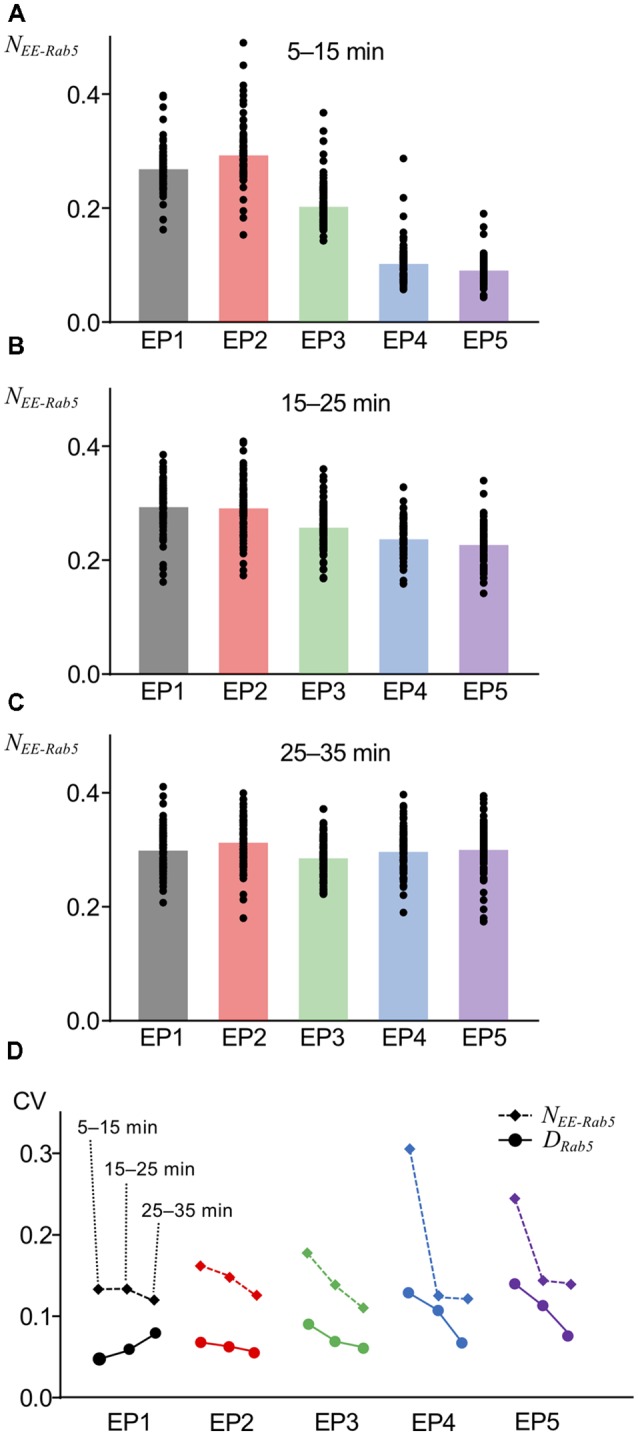
AMPAR accumulation at various timing. **(A–C)** The fraction of AMPARs coexisting with Rab5 in EE (*N_EE-Rab5_*) at 5–15 min **(A)**, 15–25 min **(B)**, and 25–35 min **(C)**. **(D)** Comparison of coefficients of variations (CVs) between *D*_Rab5_ (filled circles, solid lines) and *N*_EE-Rab5_ (filled diamonds, dashed lines). Three points from left to right show the results at 5–15 min, 15–25 min, and 25–35 min.

The variability of the number of AMPARs in endocytic vesicles was observed by electron microscopy of hippocampal neurons (Tao-Cheng et al., 2011). We included a property to the current model, namely, that the number of AMPARs internalized or leaked out is not uniform across each unit vesicle or unit site of the EE, which is a source of innate stochasticity and a crucial difference of the current model from the previous deterministic model (Kim et al., 2017). Another assumption that we included in the current model is that AMPAR localization in the vacuolar portion (the Rab5-positive portion) of the EE was independent of Rab5, and the AMPARs could spontaneously diffuse out, because to our knowledge, Rab5-dependent regulation of AMPAR localization has not been reported to date. An interesting consequence of these newly introduced variabilities in the current model was detected when AMPAR accumulation in the vacuolar portion of the EE was monitored, as has been done for Rab5 accumulation, shown in Figure 3. AMPAR accumulation was presented as the number of AMPARs coexisting with Rab5 on the EE that was normalized by basal levels of postsynaptic AMPAR number (*N_EE__-Rab5_*). Overall, averaged AMPAR accumulation in the EE appeared similar to Rab5 accumulation (Figures 4A–C). However, unlike Rab5 accumulation, AMPAR accumulation had a lower response to EP1 than EP2 during the earlier period (5–15 min) of monitoring (Figure 4A) and showed quicker accumulation of EP4 and EP5 during 15–25 min (Figure 4B), suggesting that the time course of AMPAR accumulation following application of the endocytosis profile was slightly different from Rab5 accumulation. Additionally, a comparison of the coefficients of variation (CVs) demonstrated that AMPAR accumulation had higher variability than Rab5 accumulation (Figure 4D). AMPAR accumulation monitoring in our stochastic model indicated that AMPARs mostly followed endosomal trafficking, but the distinct trafficking between AMPARs and vesicles resulted in different variability.

### Endocytosis Profile-Independent Occurrence of Sorting to LE With a Soft Threshold

**Figure 5:**
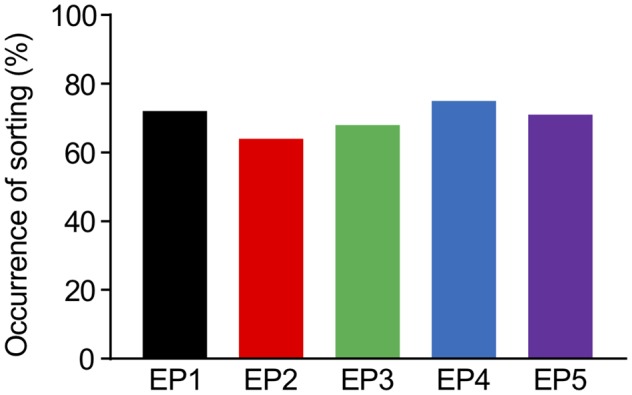
The percentages of successful occurrence of sorting from EE to LE upon the different endocytosis profiles.

In the deterministic model, the sorting from EE to LE immediately started once the accumulation of Rab5 reached the constant threshold level for sorting (Kim et al., 2017). Although a previous study modeled Rab5-Rab7 conversion as a cut-off switch with a threshold (Del Conte-Zerial et al., 2008), the experimental results implied that the conversion was actually more flexible, which may be a result of other sources of stochasticity. First, molecular interactions or reactions intrinsically contain stochasticity, which probably caused the noisy accumulation of Rab5 in the experiments. Second, the experimental results showed that it was very difficult to predict the timing of conversion, even after Rab5 accumulation appeared to be reaching saturation levels (Rink et al., 2005; Poteryaev et al., 2010). Thus, we introduced a soft threshold in the current model, which increased the probability of the sorting depending on the time of Rab5 accumulation exceeding the reference value that slightly varied around its mean value. Under conditions of this soft threshold, we observed that all endocytosis profiles had a minimum of 64% occurrence of sorting from EE to LE among the repetitions (100 times), as indicated in Figure 5. This is not a very high success rate of sorting, but considering that this model was built with high variability for a small scale (a single synapse), it appears to be sufficient to lead to multisynaptic LTD, which can usually be observed experimentally (Wang et al., 2000). An interesting part of the results was similar success rates in sorting occurrence for all endocytosis profiles (Figure 5), which might be due to the combined effects of a soft threshold with Rab5 accumulation properties, eventually reaching saturation levels, even by the dispersed endocytosis profile, as indicated in Figure 3.

### Variable Levels of LTD Maintenance Starting at Various Times

**Figure 6:**
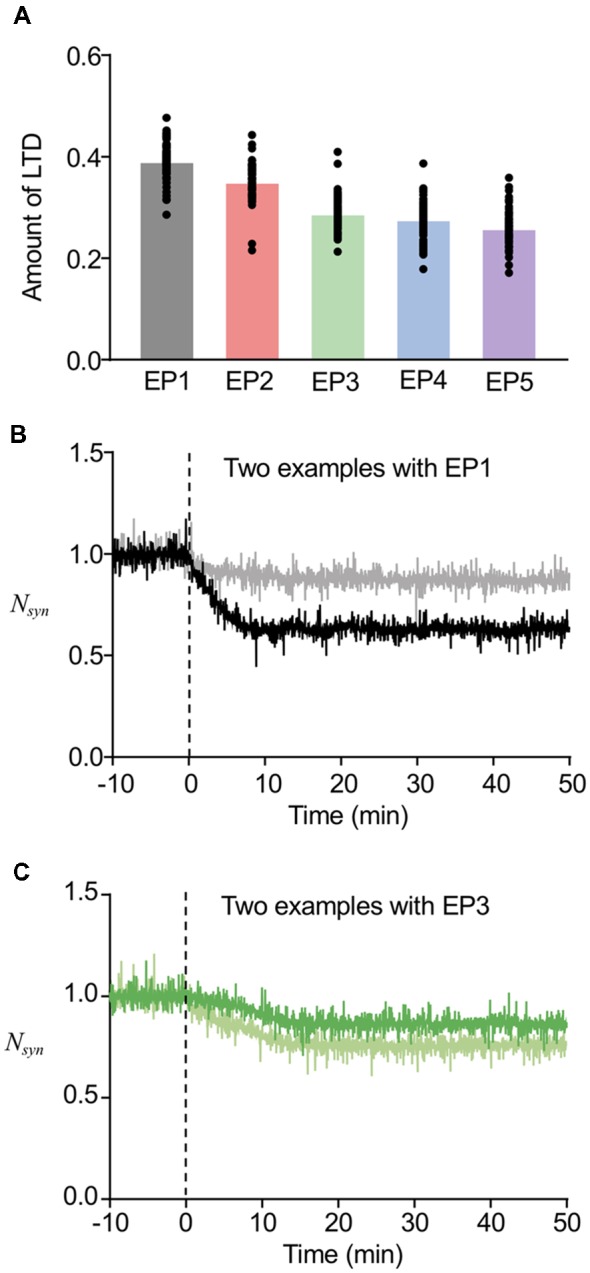
Variability in the amount of LTD. **(A)** The amount of LTD upon the different endocytosis profiles. **(B,C)** Time course of changes in *N_syn_* upon the EP1 **(B)** and EP3 **(C)**. Two example traces in the same panel showed different amount of LTD, while they showed similar timing of sorting from EE to late endosome (LE). For **(B)**, the sorting occurred at around 6 min, and for **(C)**, the sorting occurred at around 14 min.

As the current model successfully worked for both stable regulation of the number of postsynaptic AMPARs and sorting from EE to LE, we next tested whether this may also describe LTD maintenance with the involvement of sorting from EE to LE (Figure 6A). For this purpose, we only used the successful sorting examples mentioned above, because LE sorting is required for LTD maintenance (Kim et al., 2017), and first calculated the averaged *N_syn_* of these examples during 40–50 min, which indicates the maintenance level of LTD. Subtraction of the maintenance level of LTD from the basal level of *N*_syn_ was defined to be the amount of LTD. Figure 6A shows that a more focused endocytosis profile resulted in higher depression levels than dispersed profiles. This result appears to be highly relevant to the results of AMPAR accumulation shown in Figure 4. Despite the dependency of the amount of LTD on the types of endocytosis profiles, the amounts of LTD were within the range of 20%–35% on average for all endocytosis profiles, which appears to be a reasonable range, as shown in previous experimental studies (Hansel et al., 2001; Tanaka and Augustine, 2008). This suggested that we can expect LTD to be maintained, as long as EE to LE sorting occurs. In addition, the amount of LTD also varied in individual examples even when the same endocytosis profile was applied (individual data plot in Figure 6A). Examples of time courses of *N*_syn_ by EP1 (Figure 6B) and EP3 (Figure 6C) demonstrate that different amounts of LTD can be made by similar types of stimulation, whereas similar LTD amounts can be made by different types of stimulation.

**Figure 7:**
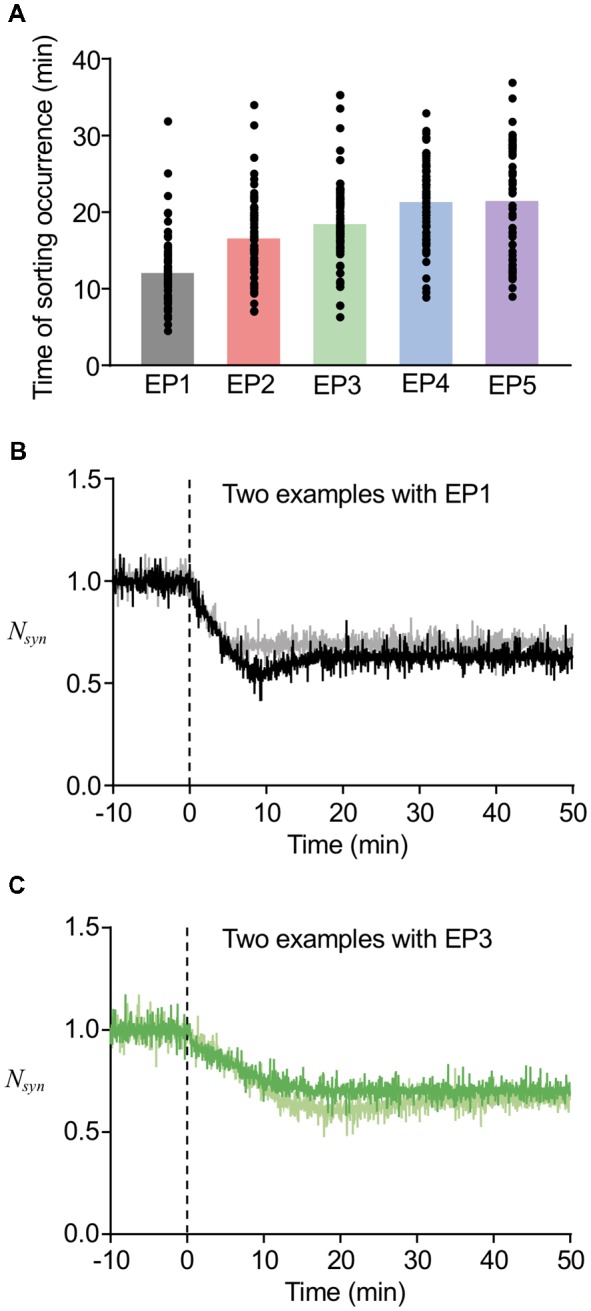
Variability in the timing of sorting occurrence. **(A)** The timing of sorting occurrence upon the different endocytosis profiles. **(B,C)** Time course of changes in *N_syn_* upon the EP1 **(B)** and EP3 **(C)**. Two example traces in the same panel showed similar amount of LTD, while they showed different timing of sorting from EE to LE. In **(B)**, the sorting occurred at 18 min for black line and at 5 min for gray line, and in **(C)** the sorting occurred at 16 min for green line and at 29 min for light green line.

In our previous study, we found that the timing of Rab5-Rab7 conversion, namely, the timing of LE sorting for LTD was varied in individual examples, and that such varied timing partially correlated with the speed of LTD expression (Kim et al., 2017). In addition, the deterministic model predicted that varying thresholds of LE sorting may be another factor of the variability in timing of LE sorting. The currently used model includes stochastic properties in endosomal and AMPAR trafficking, which appears to be reasonable based on previous experimental observations, whereas the speed of LTD expression was directly represented as the stimulus-representing endocytosis profiles EP1–EP5. The results of our new stochastic model showed that the timing of LE sorting in individual examples was highly variable regardless of the type of endocytosis profiles, yet the averaged timing of LE sorting correlated with the types of endocytosis profiles (Figure 7A). These results indicate that the current model reproduced the two characteristic properties of the timing of LE sorting during LTD, i.e., not only variability, but also partial correlation with the speed of LTD expression. These characteristic properties were observed in the LTD samples (Figures 7B,C). As the new stochastic model was able to reproduce our previous experimental results, the origin of the variability can be considered to be stochasticity in the trafficking process, including the number of AMPARs in individual vesicles or in portions of the EE, as well as threshold for LE sorting.

### Comparisons of Model Results With Experimental Results

**Figure 8:**
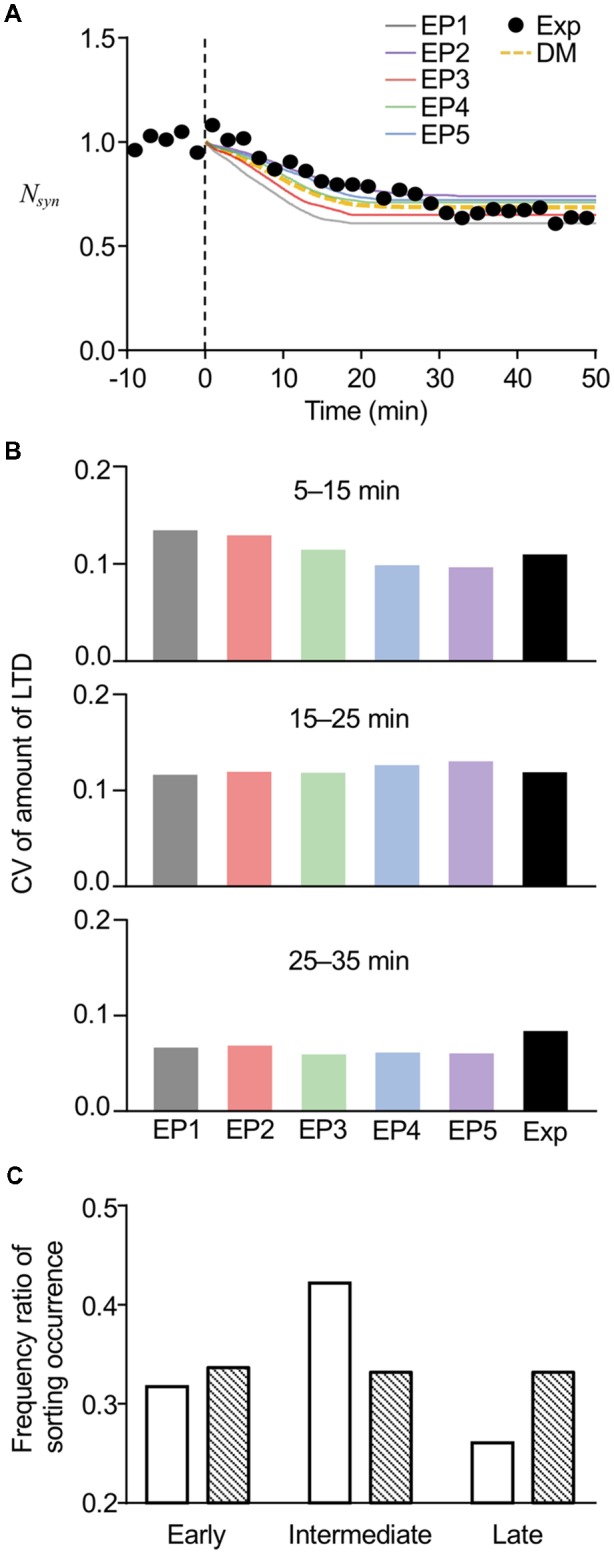
Comparisons of model results with experimental results. **(A)** Averaged time course of LTD produced by the current stochastic model (solid lines) or previous deterministic model (DM, yellow dotted line), superimposed on time course of experimentally recorded LTD (Exp, black circles). **(B)** Comparison of CVs of LTD amounts at different periods between stochastic model results and experimental results (Exp). Results of experiments were modified from data used in the previous study (Kim et al., 2017). **(C)** Comparison of frequency ratio of sorting occurrence between model results (open bars) and less variable data set (hatched bars) at early (6–13 min), intermediate (13–20 min), and late (20–27 min) periods. The less variable data set were manually generated in a way that data set had low variability yet had the same average values.

To further confirm the reproducibility of the experimental results by the current stochastic model, we directly compared the results from the model with the experimental results of LTD from our previous study. In the comparison, we also added the results obtained from our previous deterministic model. As expected from the average amount of LTD (Figure 6A) and the time of sorting occurrence (Figure 7A), the averaged time course of LTD elicited by EP1–EP5 in the present model showed different kinetics of LTD expression and a maximum level of LTD maintenance, yet the overall time course was similar to the experimental results (Figure 8A). Our previous deterministic model also produced similar time course of LTD (Figure 8A). We also calculated CVs of the amount of LTD at several time points, to quantify the variability. As expected, the CVs in the deterministic model were 0, unless we manually modified the parameters. In contrast, the CVs in the current stochastic model were approximately 0.1 at 5–15 min, similar to the experimental results (0.111, Figure 8B). The CVs at other time points were also equivalent to the experimental results (Figure 8B). Thus, whereas the deterministic model reproduced the overall time course of LTD, but not individual variability in LTD, the current stochastic model was able to reproduce both the overall time course and variability.

Even though our experimental results demonstrated that the varied timing of LE sorting partially correlated with the speed of LTD expression, the results further led to the conclusion that LE sorting occurred mostly at the intermediate time period (13–18 min), because optogenetic disturbance of LE sorting at this time prevented LTD in 75% of the cells recorded (Kim et al., 2017). To test whether the current stochastic model could reproduce this property, we plotted a histogram of sorting occurrence within the three different time periods, i.e., the early (6–13 min), intermediate (13–20 min), and late (20–27 min) time periods (Figure 8C, open bars), using the results showing LE sorting in response to EP1–EP5. Similar to our previous experimental results mentioned above Kim et al. (2017), sorting occurrence was high at the intermediate times. On the other hand, we manually generated a less variable data set with the same average values, and the histogram of the generated data showed an unbiased distribution of occurrence across the three time periods (Figure 8C, hatched bars). Thus, high variability resulting from the introduced stochasticity led to an increase in the probability of sorting occurrence at the intermediate time period, when any type of endocytosis profiles triggering LE sorting can be applied. These results imply that the stochasticity in the system helps to produce the experimental results exhibiting a relatively constant time course of LTD maintenance, despite the varied speed of LTD expression. Based on the results of this analysis, we propose that stochasticity may be linked to reliability, even though the high variabilities observed in the stochastic model would superficially give the impression that stochasticity severely harms the reliability of the system.

## Discussion

The number of postsynaptic AMPARs is stably regulated by constitutively dynamic trafficking processes. Additionally, when a postsynapse goes through a major change, such as long-term plasticity by strong stimuli, it is still able to reliably control the change of postsynaptic AMPAR numbers even though there is high variability. In this study, we extended the previously constructed cerebellar PF-PC LTD model, which included intracellular endosomal trafficking, particularly sorting from EE to LE, and built a new model including stochasticity in the trafficking process. As a result, we were able to reproduce the stable maintenance of postsynaptic AMPAR numbers both before and after LTD induction, and the variability observed in previous studies, such as the amount of LTD (Tanaka et al., 2007) and timing of sorting from EE to LE (Kim et al., 2017).

Several studies have demonstrated the involvement of endosomal trafficking in the postsynaptic regulation of AMPAR number (Gerges et al., 2004; Brown et al., 2005, 2007; Fernández-Monreal et al., 2012; Matsuda et al., 2013; Bacaj et al., 2015), and endosomal trafficking has been included in qualitative working models (Shepherd and Huganir, 2007; Langemann et al., 2008; Anggono and Huganir, 2012; Lu and Roche, 2012; Colgan and Yasuda, 2014). However, computational modeling approaches have treated endosomes as a passive component that linearly accepts and releases transported AMPARs (Earnshaw and Bressloff, 2008; Bressloff and Earnshaw, 2009; Manninen et al., 2010; Antunes and De Schutter, 2012; Czöndör et al., 2012; Gallimore et al., 2016). This idea is able to explain the relatively short time scale of synaptic plasticity and postsynaptic responses to a mild stimulus that basically enhances the recycling of AMPARs. In principle, the passive component has also been powerful to describe long-term synaptic plasticity under the assumption that the plasticity is maintained by a long-term imbalance between AMPAR internalization and reinsertion (Kuroda et al., 2001; Ogasawara and Kawato, 2009b). In reality, however, it has been shown that in cerebellar LTD, the positive feedback molecular switch leading to an imbalance is no longer required for the maintenance of LTD after a certain time (Ogasawara and Kawato, 2009a; Kim and Tanaka-Yamamoto, 2013). In our previous study, based on the experimental results showing that LE sorting is crucial for the initiation of the maintenance of LTD, we built the first model to our knowledge of postsynaptic LTD composed of AMPAR trafficking, including a nonlinearly responding endosomal component (Kim et al., 2017), namely, the Rab5-Rab7 conversion switch that controls sorting from EE to LE. This deterministic model was able to predict the source of variability, by running the simulation with varied parameter values. In our present study, we simply introduced innate stochasticity into the previous model, and were able to reproduce the high variability without affecting the trends that we observed previously. Considering that these two models are able to explain several features of cerebellar LTD, the involvement of endosomal trafficking in the regulation of postsynaptic AMPAR number should no longer be considered as a passive process, but rather needs to be included as an active controller with a stochastic nature.

As a previous study on the molecular mechanism of the Rab5-Rab7 conversion switch described (Del Conte-Zerial et al., 2008), the intracellular regulation of AMPAR number by the sorting from EE to LE appears to work as a leaky integrator that filters out high frequency noise. Comparing the PKC-MAPK positive-feedback loop switch, which integrates calcium ion flux (Kuroda et al., 2001; Tanaka et al., 2007; Tanaka and Augustine, 2008; Ogasawara and Kawato, 2009b), endosomal sorting has more complexity and integrates endocytosis more slowly. Thus, it is reasonable that the endosomal sorting switch works at a later time than the positive feedback loop switch. The difference in their functioning time scales implies that the endosomal sorting switch may filter out the fluctuation or small changes in AMPAR internalization by endocytosis, while initiating LTD maintenance. In other words, leaky integrator properties of the endosomal sorting switch enable reliable progression of LTD. In our present model, we introduced experimentally suggested stochasticity, to explain the variabilities of LTD. The important differences of the current stochastic model from the previous deterministic model are summarized as: (i) variable numbers of AMPARs in individual units of vesicles or membrane portions in the EE; (ii) diffusing out of AMPARs from the Rab5-accumulated fraction; and (iii) the soft threshold of the endosomal sorting switch. In our present model, AMPAR displacement was separated from vesicular dynamics, because of (i), and AMPAR accumulation was also separated from Rab5 accumulation because of (ii). These separations of AMPAR dynamics from typical vesicular dynamics generated the high frequency fluctuation even when there was no external stimulus. In general, a leaky integrator system accumulates inputs, yet gradually leaks small amounts of input over time. In the case of the Rab5-Rab7 conversion switch, the input is endocytosis vesicles and the leak is spontaneous diffusion of AMPARs on the EE. Because of the separation of AMPAR dynamics from vesicular dynamics in our present stochastic model, the Rab5-Rab7 conversion switch for AMPAR sorting to LE can be considered as a leaky integrator with a high amount of noise in both input of AMPAR internalization and leak of diffusing out of AMPARs. Combining the noisy leaky integrator with the soft threshold mentioned in (iii) eventually produces variable responses.

The current study demonstrated that including stochasticity in the model could clearly explain the experimentally observed variabilities, suggesting that the stochastic processes are involved in the regulation of postsynaptic AMPARs through the endosomal trafficking system. This raises the question regarding the biological advantages of the stochastic processes in AMPAR regulation. A previous theoretical study showed that synaptic efficacy fluctuations due to the stochastic exchange of AMPARs between the intracellular pool and postsynaptic receptor slots are stronger in small synapses (Triesch et al., 2018). Therefore, investigating the effects of stochastic fluctuations on LTD in synapses of different sizes is an important topic for future research.

In addition, the variability arising from the stochasticity appeared to also be beneficial for producing constant time course of LTD. As shown in Figure 8C, highly variable responses to the same stimulus eventually increased the probability of sorting within the intermediate time period, when any type of endocytosis profile triggering LE sorting could be applied. This implies that once the conditions, such as the stimulus profile and the threshold of sorting, fulfilled the requirements for successful initiation of sorting occurrence, stochasticity compensates for the variability of the stimulus profiles and reduces the variation in the timing of sorting. This phenomenon reminds us of the consequences of stochastic focusing (Paulsson et al., 2000), which indicates the beneficial effects of noise in the maintenance of LTD. Less variance in the timing of sorting also suggested the possible synchronized timing of sorting in multiple EEs. In our previous study (Kim et al., 2017), we observed two distinct responses by optogenetic disturbance of LE sorting; recovery or LTD. Considering that PF stimulation for cerebellar LTD induction is usually applied to multiple synapses due to technical difficulties in accurately stimulating a single PF, multiple EEs may be involved and some synapses may even share one EE. The two distinct responses, but not gradual and partial recovery, indicate that the sorting times from all EEs involved fall within a certain range. Even though our present model based on single synapses led to 64% sorting occurrence and consequent LTD maintenance, this synchrony in the timing of sorting of multiple EEs may result in the reliable occurrence of multisynapse LTD. This hypothesis can be evaluated by experimental studies on endosome distribution in postsynaptic areas and on LTD in single synapses, and further by a more realistic endosomal trafficking model (Vagne and Sens, 2018) of multiple synapses based on experimental observations.

## Data Availability

The datasets for this manuscript are available from the corresponding authors upon reasonable request. The model script is available at https://sites.google.com/view/closeyes.

## Author Contributions

TK built the model and performed simulation. Both authors designed the model and wrote the article.

## Conflict of Interest Statement

The authors declare that the research was conducted in the absence of any commercial or financial relationships that could be construed as a potential conflict of interest.
